# A multi‐omic biomarker signature in pre‐treatment rectal tumours stratifies patients with different pathological responses to neoadjuvant treatment

**DOI:** 10.1002/ctm2.70576

**Published:** 2025-12-26

**Authors:** Laura E. Kane, Croí E. Buckley, Rebecca M. O'Brien, Meghana S. Menon, Aisling B. Heeran, Xiaofei Yin, Timothy S. Nugent, Noel E. Donlon, John V Reynolds, Adnan Hafeez, Diarmuid S. O'Ríordáin, Robert A. Hannon, Paul Neary, Reza Kalbassi, Brian J. Mehigan, Paul H. McCormick, Cara Dunne, John O. Larkin, Lorraine Brennan, Michael E. Kelly, Jacintha O'Sullivan, Niamh Lynam‐Lennon

**Affiliations:** ^1^ Department of Surgery School of Medicine Trinity Translational Medicine Institute Trinity College Dublin Dublin Ireland; ^2^ Trinity St. James's Cancer Institute St. James's Hospital Trinity College Dublin Dublin Ireland; ^3^ School of Pharmacy and Biomolecular Sciences RCSI University of Medicine and Health Sciences Dublin Ireland; ^4^ UCD School of Agriculture and Food Science UCD Institute of Food and Health Conway Institute University College Dublin Dublin Ireland; ^5^ Department of Surgery Beacon Hospital Dublin Ireland; ^6^ Gastrointestinal Medicine and Surgery (GEMS) Directorate St. James's Hospital Dublin Ireland; ^7^ Department of Biology Kathleen Lonsdale Institute for Human Health Research Maynooth University Co. Kildare Ireland

**Keywords:** biomarkers, metabolomics, rectal cancer, therapy response, transcriptomics

1

Dear Editor,

Rectal cancer (RC) incidence is rising, particularly in individuals < 50 years, who present with aggressive, treatment‐refractory tumours.^1^ Resistance to neoadjuvant treatment (neo‐tx) is a significant problem, with no biomarkers of response currently in use. Tumours of similar clinical characteristics can have vastly different responses to neo‐tx, suggesting the dichotomy in response is due to differences in the tumour molecular environment. Alterations in mitochondrial function and energy metabolism play a role in the pathogenesis of gastrointestinal cancers,^2^, ^3^ implicating the metabolome as a potential untapped source of predictive biomarkers. To address this unmet need, we performed multi‐omic analysis of metabolomic and transcriptomic profiles from normal, non‐cancer rectal tissue and pre‐treatment RC biopsies (Supporting Information) to identify alterations associated with the pathogenesis of RC.

Liquid chromatography‐mass spectrometry revealed 29 metabolites significantly altered in RC tissue (*n *= 32) compared to non‐cancer rectal tissue (*n *= 20) (Figure 1A). Pathway analysis uncovered 65 upregulated and four downregulated pathways significantly associated with altered metabolites (Figure 1B). Most altered metabolites were lipid molecules or mediators of lipid metabolism, suggesting that remodelling of lipid metabolism is a feature of RC. Diacyl phosphatidylcholines (PCs) are important mediators of lipid metabolism, supporting other studies highlighting a role for choline metabolism and lipid remodelling in tumourigenesis.^4^ SM C18:0 and SM (OH) C22:1 are sphingolipids, important structural lipid components of biological membranes, which support the physiological function of the colon and are deregulated in RC.^5^ Interestingly, cancer cells hydrolyse sphingomyelin to maintain production of PCs,^4^ suggesting a mechanism for the concomitant increase in PCs and decrease in sphingomyelins demonstrated in RC here.

**FIGURE 1:**
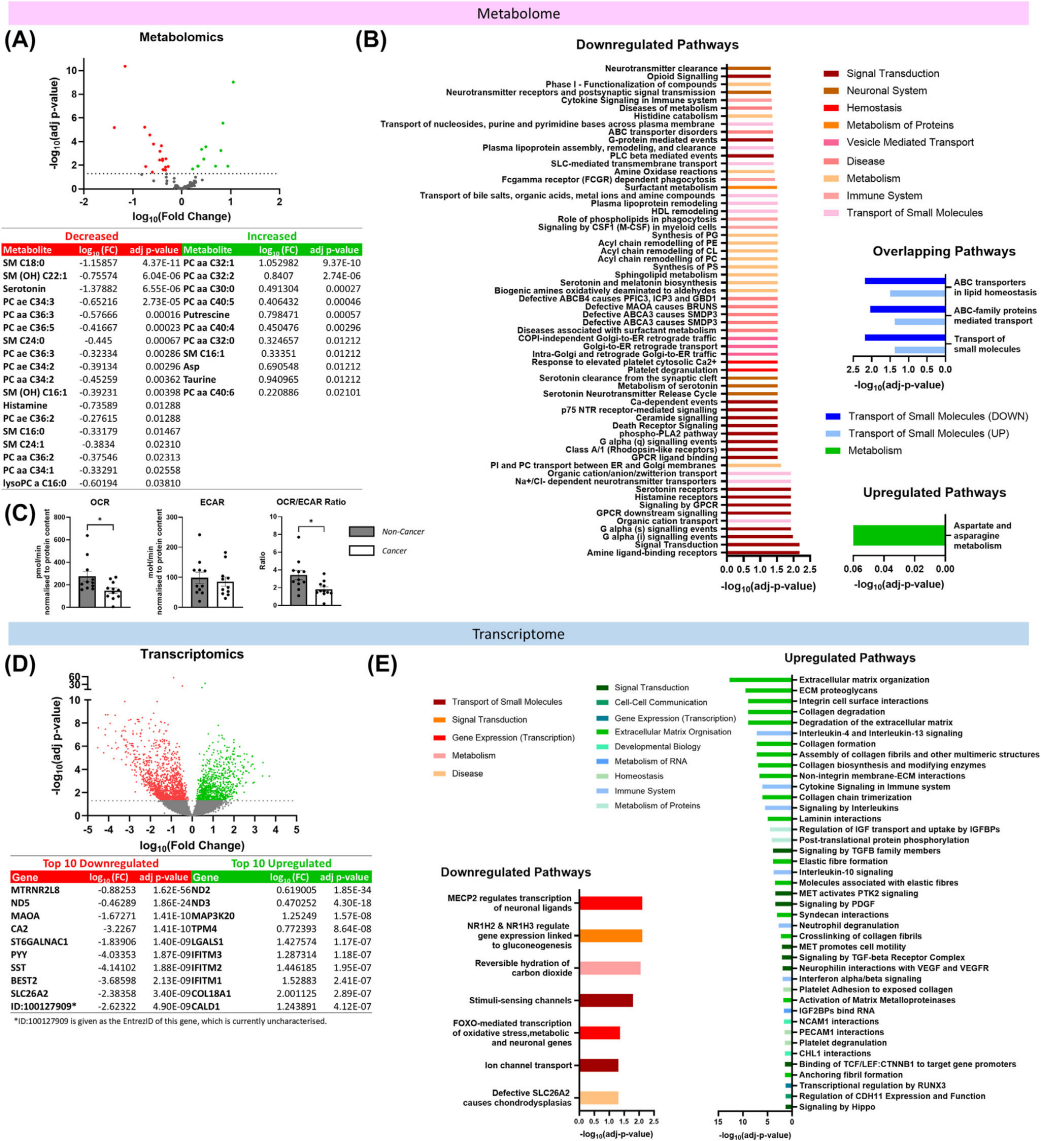
**The metabolome and transcriptome of rectal tumours are distinct from those of normal non‐cancer rectal tissue**. (A) Differential expression analysis comparing the metabolome of non‐cancer rectum (*n *= 20) and rectal cancer tissue (*n *= 32) (*p* < 0.05, false discovery rate [FDR] = 0.05); 18 decreased and 11 increased metabolites in rectal cancer (RC) compared to normal non‐cancer tissue. (B) Reactome pathway analysis of significantly altered metabolites. (C) Basal metabolic phenotyping of pre‐treatment rectal cancer biopsies (*n *= 11) and non‐cancer rectal tissue biopsies (*n *= 12) as assessed via Seahorse Biosciences XFe24 analyser. OCR = Oxygen Consumption Rate, a measure of oxidative phosphorylation; ECAR = Extracellular Acidification Rate, a measure of glycolysis. Data are presented as mean ± SEM. Statistical analysis was performed by the Wilcoxon test or Mann‐Whitney U test as appropriate (*p < 0.05, **p < 0.01, and ***p < 0.001). (D) Differential expression analysis comparing the transcriptome of non‐cancer rectum (*n *= 28) and rectal cancer tissue (*n *= 31) (*p* < 0.05, FDR = 0.05); 919 upregulated and 1418 downregulated in RC compared to normal non‐cancer tissue. (E) Reactome pathway analysis of significantly altered genes.

Real‐time metabolic analysis demonstrated that (Figure 1C)OCR rates and OCR/ECAR ratios were significantly decreased in RC compared to non‐cancer rectal tissue, highlighting metabolic remodelling in RC. Inhibition of mitochondrial metabolism results in accelerated turnover of PCs in neuronal cells,^6^ suggesting a mechanism underlying the altered choline metabolism demonstrated in RC.

Transcriptomics revealed 2337 genes differentially expressed between RC (*n *= 31) and non‐cancer rectal tissue (*n *= 28) (Figure 1D). Pathway analysis revealed 41 upregulated and seven downregulated pathways significantly associated with altered genes (Figure 1E). Interestingly, several of the most altered genes play roles in mitochondrial respiration. *ND2*, *ND3* and *ND5* encode subunits of the NADH dehydrogenase enzyme, a crucial component in the electron transport chain, supporting the altered OCR demonstrated in RC tissue.

In pre‐treatment RC biopsies, altered metabolites had significant correlations with OCR, ECAR, OCR/ECAR ratios, and several clinical variables, including the modified Ryan Tumour Response Score (TRS) (TRS0 = complete response; TRS1 = near complete response; TRS2 = partial response) (Figure 2A). Two metabolites correlated significantly with TRS: Serotonin and lysoPC a C16:1 (Figure 2B). Unsupervised hierarchical clustering showed decent grouping in TRS2, with little distinction between TRS0 and TRS1, suggesting similar expression profiles between the two (Figure 2C). Only lysoPC a C16:1 had significant correlations with clinical variables (Figure 2D).

**FIGURE 2:**
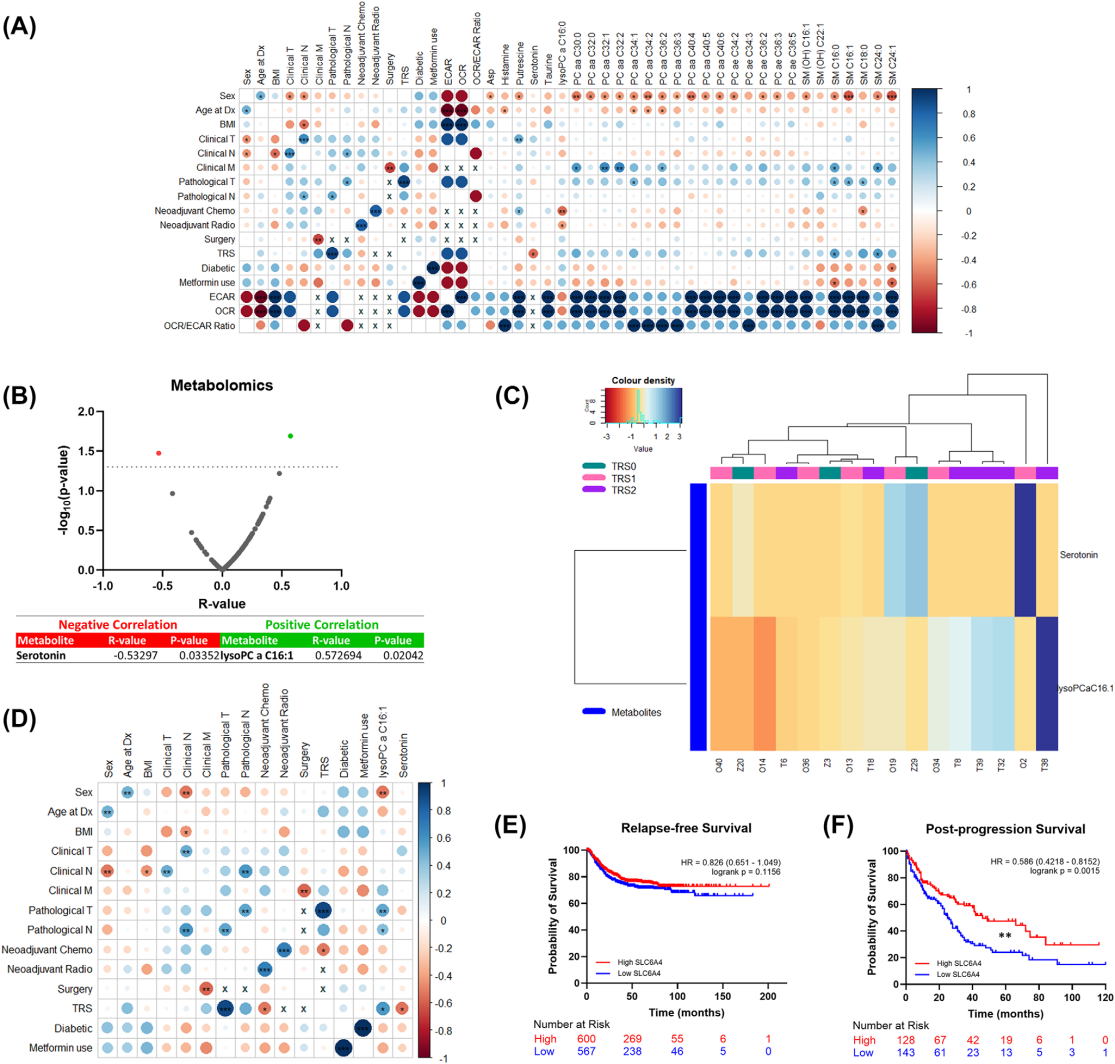
**Expression of two metabolites in rectal cancer tissue significantly correlates with Tumour Response Score (TRS)**. (A) Correlations between patient clinical data, differentially expressed metabolites, and metabolic outcomes of the Seahorse Biosciences XFe24 analyser are given as a corrplot. Colour intensity relates to R value, circle size relates to the *p*‐value (*p < 0.05, **p < 0.01, and ***p < 0.001). ‘x’ indicates that there was not sufficient data to correlate these variables. (B) Correlation analysis of the metabolome of rectal cancer tissue and TRS (*p* < 0.05). (C) Unsupervised hierarchical clustering of patients into TRS groups based on their expression of the two highly correlated metabolites. Dendrograms show (top) the relatedness of the patients, and (left) the relatedness of the metabolites. (D) Correlations between patient clinical data and highly correlated metabolites are given as a corrplot. Colour intensity relates to R value, circle size relates to the *p*‐value (*p < 0.05, **p < 0.01, and ***p < 0.001). ‘x’ indicates that there was not sufficient data to correlate these variables. Kaplan‐Meier curves comparing colorectal cancer patient (E) relapse‐free survival and (F) post‐progression survival rates with low and high SLC6A4 (serotonin) mRNA expression (**p < 0.01).

*SLC6A4* expression in CRC tissue, which transcribes serotonin, had no significant effect on relapse‐free survival (Figure 2E), while low *SLC6A4* resulted in significantly worse post‐progression survival (Figure 2F). Serotonin is demonstrated to enhance radiosensitivity in colon cancer,^7^ suggesting that the decreased serotonin in TRS2 patients is a mechanism underlying neo‐tx resistance.

Altered genes had significant correlations with several clinical variables (Figure 3A). *RPL30* and *CXCL14* were significantly upregulated, and *SNORA81*, *SNORD50A*, *LCN2* and *SNORA64* were significantly downregulated in TRS2 compared to TRS0 (Figure 3B). Increased expression of *RPL30* is associated with amplification of the oncogene *MYC*, which promotes cytotoxic therapy resistance.^8^ Deletion of *SNORD50A* is associated with poorer survival outcomes in several cancers.^9^ Interestingly, *SNORD50A* binds and inhibits the oncogene *KRAS*, with depletion of *SNORD50A* causing activation of the MAPK cascade,^9^ which is involved in tumour resistance to therapy,^10^ suggesting a potential role in neo‐tx resistance in RC.

**FIGURE 3:**
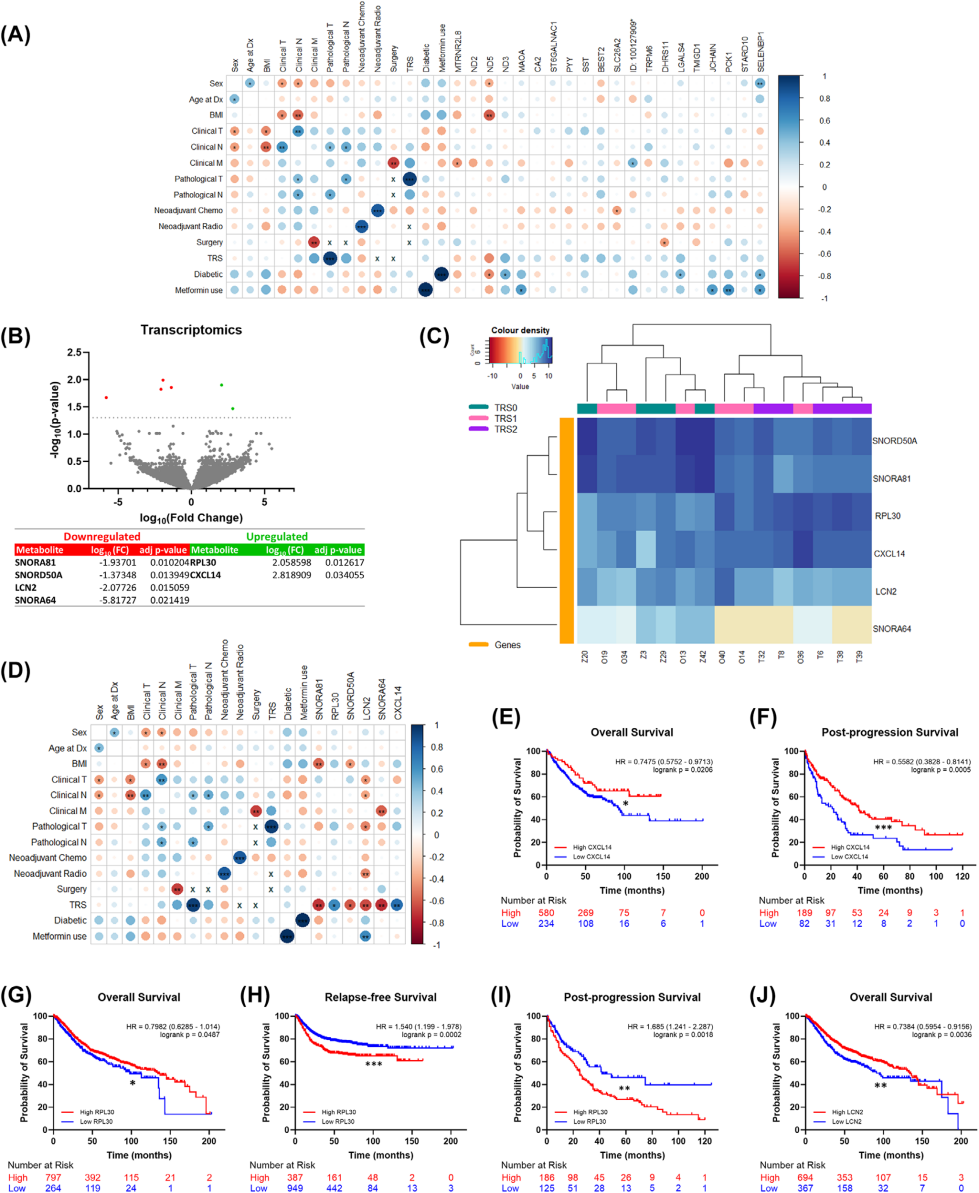
**Expression of six genes is significantly altered in rectal cancer tissue from patients having poorer pathological responses to neo‐tx**. (A) Correlations between patient clinical data and differentially expressed genes are given as a corrplot. Colour intensity relates to R value, circle size relates to the p‐value (*p < 0.05, **p < 0.01, and ***p < 0.001). ‘x’ indicates that there was not sufficient data to correlate these variables. (B) Differential expression analysis of the transcriptome of rectal cancer tissue from patients having a complete pathological response (TRS0) and partial pathological response (TRS2) (*p *< 0.05). (C) Unsupervised hierarchical clustering of patients into Tumour Response Score (TRS) categories based on their expression of the six differentially expressed genes. Dendrograms show (top) the relatedness of the patients, and (left) the relatedness of the genes. (D) Correlations between patient clinical data and differentially expressed genes are given as a corrplot. Colour intensity relates to R value, circle size relates to the p‐value (*p < 0.05, **p < 0.01, and ***p < 0.001). ‘x’ indicates that there was not sufficient data to correlate these variables. Kaplan‐Meier curves comparing CRC patient (E) overall survival and (F) post‐progression survival rates with low and high CXCL14 mRNA expression. Kaplan‐Meier curves comparing CRC patient (G) overall survival, (H) relapse‐free survival and (I) post‐progression survival rates with low and high RPL30 mRNA expression. Kaplan‐Meier curve comparing CRC patient (J) overall survival rates with low and high LCN2 mRNA expression (*p < 0.05, **p < 0.01, and ***p < 0.001).

Separation of TRS based on gene expression is improved, with a clear distinction between TRS0 and TRS2 (Figure 3C). Several genes had significant correlations with clinical variables (Figure 3D). Low *CXCL14* expression was associated with significantly worse overall (Figure 3E) and post‐progression survival (Figure 3F). High *RPL30* was associated with significantly longer overall survival (Figure 3G); however, it resulted in significantly worse relapse‐free survival (Figure 3H) and post‐progression survival (Figure 3I), aligning with the high *RPL30* in TRS2 patients. Similarly, low *LCN2* expression was associated with worse overall survival (Figure 3J).

Altered genes and metabolites were integrated into an 8‐feature multi‐omic biomarker panel. Only TRS0 (*n *= 3), TRS1 (*n *= 6) and TRS2 (*n *= 5) groups could be included in the final analysis as matched transcriptomic data were not available for the TRS3 patient. TRS0 and TRS2 cluster separately using these eight features, with TRS1 interspersed between them (Figure 4A). Principal component analysis showed similar patterns, with Serotonin, *SNORA64*, *SNORD50A*, *SNORA81*, *RPL30* and *CXCL14* contributing most to group separation (Figure 4B). Examining expression of each feature individually, TRS0 and TRS2 have higher expression levels of separate features, while TRS1 sits within the range of TRS0 and TRS2 (Figure 4C). Leave‐one‐out cross‐validation demonstrated poor distinction of TRS0 from TRS1/TRS2 (area under the curve [AUC] = 0.273, Sensitivity = 0%, Specificity = 54.5%), likely due to an imbalance for samples between the groups (TRS0 = 3, TRS1/TRS2 = 11) and thus not accurately representing the panel's performance between these groups (Figure 4D). Classification accuracy for TRS0 versus TRS2 was far superior (AUC = 0.933, Sensitivity = 100%, Specificity = 80%), aligning with the distinct expression profiles observed (Figure 4B,C). Lastly, in this pilot cohort, TRS0/TRS1 versus TRS2 demonstrated perfect classification (AUC = 1, Sensitivity = 100%, Specificity = 100%), suggesting that overall, the expression profiles of TRS2 patients are distinct from both TRS0 and TRS1, which are more closely related to each other (Figure 4D).

**FIGURE 4:**
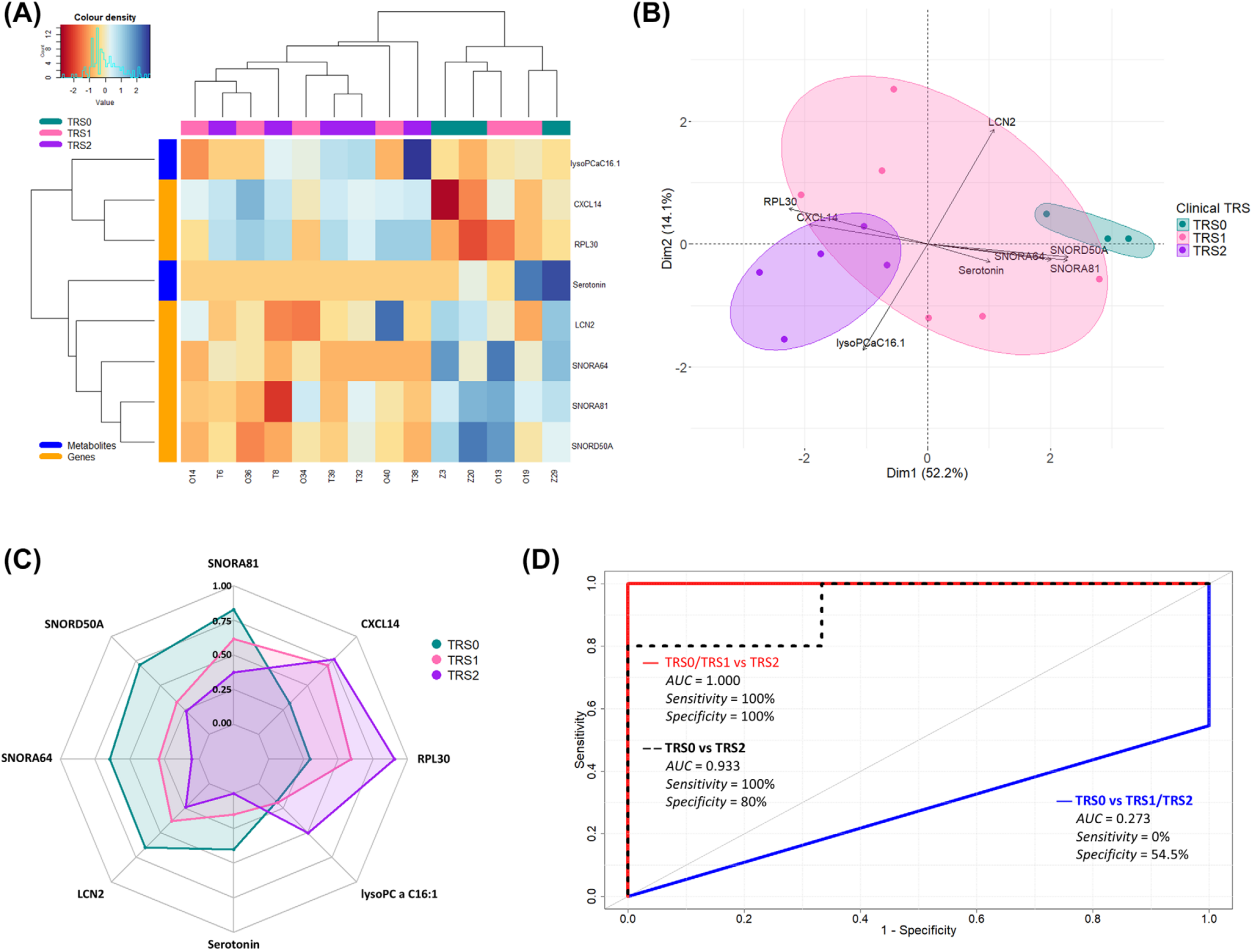
**8‐feature multi‐omic panel predicts TRS2 from TRS0 and TRS1 with perfect accuracy**. (A) Unsupervised hierarchical clustering of patients into Tumour Response Score (TRS) groups based on their expression of the 8 features. Dendrograms show (top) the relatedness of the patients, and (left) the relatedness of the features. (B) 2D Principal component analysis using the 8‐feature multi‐omic panel, with biplot overlayed. Biplot scale is set to zero to ensure vectors (arrows) are scaled to represent their respective loadings. The length of each vector is proportional to the variance of the corresponding feature. (C) Radar chart showing the scaled, mean value for each feature across the TRS groups. (D) ROC Curves generated from leave‐one‐out cross validations of the 8‐feature panel comparing various combinations of TRS groups.

In a pilot cohort, we demonstrate that rectal tumours have metabolic and transcriptomic remodelling, highlighting altered lipid metabolism as a common feature in rectal tumours, suggesting a novel therapeutic targeting approach. We also identify a novel multi‐omic 8‐biomarker panel, with potential, following validation in an independent cohort representing the full TRS spectrum, as a predictive signature of pathological response to neo‐tx for improved patient stratification.

## AUTHOR CONTRIBUTIONS

**Laura E. Kane**: Data curation, Formal analysis and Writing ‐ original draft. **Croí E. Buckley**: Investigation, Data curation and Formal analysis. **Rebecca M. O.Brien**: Data curation and Resources. **Meghana S. Menon**: Data curation and Resources. **Aisling B. Heeran**: Data curation and Resources. **Xiaofei Yin**: Investigation and Formal analysis. **Timothy S. Nugent**: Clinical samples and Clinical data. **Noel E. Donlon**: Clinical samples and Clinical data. **John V. Reynolds**: Resources. **Adnan Hafeez**: Clinical samples. **Diarmuid S. O.R‐ordáin**: Clinical samples. **Robert A. Hannon**: Clinical samples. **Paul Neary**: Clinical samples. **Reza Kalbassi**: Clinical samples. **Brian J. Mehigan**: Clinical samples. **Paul H. McCormick**: Clinical samples. **Cara Dunne**: Clinical samples. **John O. Larkin**: Clinical samples. **Lorraine Brennan**: Formal analysis and Writing ‐ review and editing. **Michael E. Kelly**: Clinical samples, Clinical data and Writing ‐ review and editing. **Jacintha O. Sullivan**: Conceptualization, Supervision and Writing ‐ review and editing. **NLL**: Conceptualization, Supervision, Funding acquisition, Writing. original draft and Writing ‐ review and editing.

## CONFLICT OF INTEREST STATEMENT

The authors declare no conflict of interest.

## FUNDING INFORMATION

This research was funded by the Health Research Board (Ireland), grant number: EIA‐2017‐020. We also acknowledge funding from The Comprehensive Molecular Analytical Platform (CMAP) under the SFI Research Infrastructure Programme, reference number: 18/RI/5702. LE. Kane is supported by a Research Ireland fellowship, grant number: GOIPD/2024/331

## ETHICS STATEMENT

This research was conducted in compliance with the Declaration of Helsinki and was approved by the joint St James's Hospital/AMNCH ethical review board (Ref: 2011/43/02) and the Beacon Hospital Research Ethics Committee (Reference BEA0139).

## PATIENT CONSENT STATEMENT

Informed consent was obtained from all subjects involved in the study.

## Supporting information



Supporting Information

## Data Availability

Detailed data are available from the corresponding author on reasonable request.

## References

[1] Sung H , Ferlay J , Siegel RL , et al. Global Cancer Statistics 2020: GLOBOCAN estimates of incidence and mortality worldwide for 36 cancers in 185 countries. CA Cancer J Clin. 2021;71(3):209‐249. doi:10.3322/caac.21660 33538338

[2] Buckley CE , Yin X , Meltzer S , et al. Energy metabolism is altered in radioresistant rectal cancer. Int J Mol Sci. 2023;24(8):7082. doi:10.3390/ijms24087082 37108244 PMC10138551

[3] Lynam‐Lennon N , Maher SG , Maguire A , et al. Altered mitochondrial function and energy metabolism is associated with a radioresistant phenotype in oesophageal adenocarcinoma. PLoS One. 2014;9(6):e100738. doi:10.1371/journal.pone.0100738 24968221 PMC4072695

[4] Saito RF , Andrade LNS , Bustos SO , Chammas R . Phosphatidylcholine‐derived lipid mediators: the crosstalk between cancer cells and immune cells. Front Immunol. 2022;13:768606. doi:10.3389/fimmu.2022.768606 35250970 PMC8889569

[5] Markowski AR , Blachnio‐Zabielska AU , Pogodzinska K , Markowska AJ , Zabielski P . Diverse sphingolipid profiles in rectal and colon cancer. Int J Mol Sci. 2023;24(13):10867. doi:10.3390/ijms241310867 37446046 PMC10341971

[6] Farber SA , Slack BE , Blusztajn JK . Acceleration of phosphatidylcholine synthesis and breakdown by inhibitors of mitochondrial function in neuronal cells: a model of the membrane defect of Alzheimer's disease. FASEB J. 2000;14(14):2198‐2206. doi:10.1096/fj.99-0853 11053240

[7] Curtis JJ , Vo NTK , Seymour CB , Mothersill CE . 5‐HT(2A) and 5‐HT(3) receptors contribute to the exacerbation of targeted and non‐targeted effects of ionizing radiation‐induced cell death in human colon carcinoma cells. Int J Radiat Biol. 2020;96(4):482‐490. doi:10.1080/09553002.2020.1704911 31846381

[8] de Mey S , Dufait I , De Ridder M . Radioresistance of human cancers: clinical implications of genetic expression signatures. Front Oncol. 2021;11:761901. doi:10.3389/fonc.2021.761901 34778082 PMC8579106

[9] Siprashvili Z , Webster DE , Johnston D , et al. The noncoding RNAs SNORD50A and SNORD50B bind K‐Ras and are recurrently deleted in human cancer. Nat Genet. 2016;48(1):53‐58. doi:10.1038/ng.3452 26595770 PMC5324971

[10] Lee S , Rauch J , Kolch W . Targeting MAPK signaling in cancer: mechanisms of drug resistance and sensitivity. Int J Mol Sci. 2020;21(3):1102. doi:10.3390/ijms21031102 32046099 PMC7037308

