# Supplementary material for: A multi‐omic biomarker signature in pre‐treatment rectal tumours stratifies patients with different pathological responses to neoadjuvant treatment
Source: Clin Transl Med. 2025 Dec 26;16(1):e70576. doi: 10.1002/ctm2.70576 (PMC12743138; doi:10.1002/ctm2.70576)
Supplement: Supplementary file 1 — Supporting Information [file CTM2-16-e70576-s001.docx]

Supplementary material

Materials and Methods

***Ethical approval and sample collection***

Ethical approval for patient sample collection was granted by the joint St James's Hospital/AMNCH ethical review board (Ref: 2011/43/02) and the Beacon Hospital Research Ethics Committee (Reference BEA0139). Patients undergoing colonoscopy were recruited between January 2018 and October 2021 at St. James’s Hospital, Dublin and between October 2020 and January 2022 at the Beacon Hospital, Dublin. Following written informed consent, rectal tissue was obtained by a qualified endoscopist from patients during colonoscopy. Pre-treatment tumour biopsies were obtained from consenting rectal cancer patients. Normal (non-cancer) rectal biopsies were obtained from consenting patients undergoing colonoscopy for gastrointestinal symptoms, including unexplained rectal bleeding and changes in bowel habit, who did not have cancer. All tissue biopsy samples were assessed by an experienced gastrointestinal pathologist and histologically confirmed using haematoxylin and eosin staining to be tumour or non-tumour tissue.

***Patient treatment and response to neoadjuvant treatment***

Rectal cancer patients from St. James’s Hospital or the Beacon Hospital received either neo-CRT, neoadjuvant radiation therapy (neo-RT), neoadjuvant chemotherapy (neo-CT), surgery only, or chemotherapy (CT) only. CT consisted of FOLFOX (leucovorin, 5-FU, oxaliplatin) or FOLFIRI (leucovorin, 5-FU, irinotecan hydrochloride). RT was delivered in 28 fractions of 1.8 Gy.

Response to neoadjuvant treatment (neo-tx) was assessed pathologically using resected rectal specimens. Tumour regression score was determined using the modified Ryan tumour regression score (TRS), which uses the ratio of fibrosis to residual tumour, where TRS0 (a complete response) is defined as no viable tumour cells, TRS1 (a near-complete response) refers to single or small groups of cancer cells, TRS2 (a partial response) describes tumour regression that is evident to a greater extent than single or groups of cancer cells and TRS3 (a poor or no response) defines residual cancer that is extensive, with no evidence of tumour regression ^1^.

***Metabolomic Profiling***

***Preparation of patient samples for metabolomic analysis***

Frozen tissue was weighed and grinded with liquid nitrogen followed by addition of ice-cold extraction solvent (Ethanol: PBS = 85:15). The samples were subsequently centrifuged at 10000 × *g* for 5 min at 4°C. The supernatant was collected and stored at -80°C for metabolite measurement.

***AbsoluteIDQ^®^ p180 assay***

Metabolites were identified and quantified using the AbsoluteIDQ^®^ p180 assay (Biocrates Life Sciences, Innsbruck, Austria) according to the manufacturers’ instructions. Detailed sample preparation and analysis were previously described ^2^. Briefly, 10 µL of sample supernatant from serum and tissue were added to the 96-well plate and dried under a stream of nitrogen. A total of 50 µL of 5% phenyl isothiocyanate solution was added to each well and incubated for 25 min at room temperature. Following incubation, the plate was dried for 60 min. The extraction solvent (5 mM ammonium acetate in methanol, 300 µL) was added to each well and the plate was subsequently incubated for 30 min with shaking. The plate was centrifugated at 500 × *g* for 2 min to obtain the eluate, and 150 µL of eluate was diluted with 150 µL of HPLC grade water for liquid chromatography-tandem mass spectrometry (LC-MS/MS) run. A total of 50 µL of eluate was diluted with 450 µL mobile phase for the flow injection analysis-tandem mass spectrometry (FIA-MS/MS) run.

The data were acquired on a SCIEX QTRAP 6500plus mass spectrometer coupled to SCIEX ExionLC™ Series UHPLC capability. During LC-MS/MS run, a UHPLC column provided with the AbsoluteIDQ® p180 kit was installed for metabolite separation, and water and acetonitrile (with 0.2% formic acid) were used as mobile phase A and B, respectively. Amino acids (*n*=21) and biogenic amines (*n*=21) were identified and quantified in positive mode. For the FIA-MS/MS analyses, methanol was employed as the running solvent, and 40 acylcarnitines, 14 lysophosphatidylcholines (lysoPC), 38 acyl/acyl phosphatidylcholines (PC aa), 38 acyl/alkyl phosphatidylcholines (PC ae), 15 sphingomyelins (SMs), and the sum of hexoses (H1) were identified and semi-quantified in positive mode. In this assay, all metabolites were analyzed using multiple reaction monitoring (MRM). Data acquisition was conducted by the software of AB Sciex Analyst^®^ version1.7.2.

***Metabolomic data processing and metabolite quantification***

Amino acids and biogenic amines were quantified based on isotopically labelled internal standards and 7-point calibration curves using AB Sciex Analyst^®^ version1.7.2 software. Other metabolites, such as acylcarnitines, lysoPCs, PCs, SMs and hexose were semi-quantified by using 14 internal standards in the MetIDQ™ software (Biocrates Life Sciences). Data quality was evaluated by checking the accuracy and reproducibility of QC samples. Finally, the concentrations of metabolites were reported in µM. For further statistical analyses, metabolites were included only when the concentrations of metabolites were above the limit of detection (LOD) in more than 50% of samples.

***Real time metabolic profiling of tissue***

Tissue biopsies were collected at colonoscopy, placed on saline-soaked gauze, and transported to the laboratory. Each biopsy was placed into an individual well of an XF24 Islet Capture Microplate (Agilent Technologies) and secured by islet capture screens. A volume of 1 mL complete M199 (Gibco) (supplemented with foetal bovine serum (FBS) (10%), penicillin-streptomycin (1%), Fungizone™ (1%), gentamycin (0.1%) and insulin (1 µg/mL)) was placed in each well. The plate was placed at 37°C, in 5% CO_2_/95% humidified air for 30 min to equilibrate.

Three basal measurements of OCR and ECAR were measured over 24 min, consisting of three repeats of mix (3 min)/ wait (2 min) / measurement (3 min) using the Seahorse XFe24 analyser, as described previously ^3^. Protein was isolated from patient tissue biopsies using the AllPrep DNA/RNA/Protein Mini Kit, according to manufacturers’ instructions. Metabolic rates were normalised to protein content using the bicinchoninic acid (BCA) protein assay (Pierce, Thermo Fisher), according to the manufacturer’s instructions.

***RNA isolation and quantification from biopsies***

Total RNA was isolated from tissue biopsies using the AllPrep DNA/RNA/Protein Mini Kit (Qiagen), according to manufacturers’ instructions. To quantify the RNA, a Nanodrop 1000 spectrophotometer (version 3.1, Nanodrop technology) was utilised.

***Transcriptomic Profiling***

Transcriptomic profiling was conducted utilizing mRNA sequencing using the Lexogen QuantSeq 3′ mRNA-Seq. RNA samples were prepared for sequencing using the QuantSeq™ 3′ mRNA-Seq Library prep kit (Lexogen), according to the manufacturer’s instructions, using a starting volume of 50 ng RNA. An equal molar amount of the purified library was pooled for sequencing, with a loading concentration of 320 pM loaded onto the NovaSeq flowcell. Sequencing was performed using the NovaSeq 6000 (Illumina, San Diego, CA, USA) and an SP v1.5 sequencing kit (Illumina) with 1 × 100 bp reads, as per the manufacturer’s instructions.

***Transcriptomic mapping***

Raw sequencing data were received as forward and reverse FASTQ files for each sample. FASTQ files were quality checked using the fastQC tool, before being trimmed as paired reads using the Trimmomatic tool ^4^. Samples with low sequencing quality were excluded from downstream analyses. The reference human genome *hg19* was built in RStudio using the Bioconductor package ‘Rsubread’ (v2.16.0). Trimmed FASTQ files were loaded into RStudio, and all downstream analyses were carried out in this software. The trimmed FASTQ files were mapped to the reference genome using the *align* function in the ‘Rsubread’ package, and mapped reads were produced as BAM files. BAM files were assigned to genomic features using the *featureCounts* function of the ‘Rsubread’ package, to obtain the number of reads mapped per gene. Gene annotations for the RNAseq EntrezGene IDs were obtained using the *ann* function in the Bioconductor package ‘org.Hs.eg.db’ (v3.18.0). Counts per million (CPM) were generated using the ‘edgeR’ package (v4.0.3), and CPM data were filtered to include only genes with more than 1 million CPM reads in at least 4 samples.

***Pathway Mapping***

Altered genes and metabolites were entered into the Reactome Pathway Browser (version 3.7) analysis tool as either an upregulated or downregulated database ^5^. Significantly enriched pathways (FDR<0.05) were sorted under the platform’s hierarchy and exported into GraphPad Prism (v10.4.0) for visualisation.

***Data Mining***

The Kaplan-Meier plotter was used to examine the effect of low and high mRNA expression on the overall survival, relapse-free and progression-free survival of patients with CRC ^6^. To select the expression cut-off between the groups, all possible cut-off values between the lower and upper quartiles were computed, with the best performing cut-off being selected. Cox proportional hazards regression analysis was performed to assess the relationship between mRNA expression levels and survival. Data were exported into GraphPad Prism (v10.4.0) for visualisation.

***Statistical Analysis***

All graphing was performed using Graphpad Prism v9 software and R Studio (v21.09.0). Data are presented as mean ± standard error of the mean (SEM) throughout. Statistical comparisons were carried out using Mann-Whitney U test, as appropriate and described in figure legends. The R code used for these analyses can be found at github.com/LauraKanePhD. Differentially expressed genes were identified in R Studio using empirical Bayes statistics for differential expression and Benjamini & Hochberg multiple comparisons adjustment from the ‘limma’ package (v3.58.1). Volcano plots of significantly differentially expressed factors, and bar charts depicting Reactome pathway analysis were created in GraphPad Prism (v10.4.0). Corrplots illustrating the correlations between patient clinical data and omics factors, and Spearman correlations of metabolic data and TRS, were created in R Studio using packages ‘Hmisc’ (v4.7-2) and ‘corrplot’ (v0.90). Unsupervised hierarchical clustering (UHC) with supporting heatmap and dendrograms were generated in R Studio using packages ‘edgeR’ (v3.32.1), ‘cluster’ (v2.1.4), ‘purrr’ (v0.3.4), ‘dendextend’ (v1.15.2), ‘dplyr’ (v1.0.9), ‘ggplot2’ (v3.3.5), ’ComplexHeatmap’ (v2.6.2), ‘RColorBrewer’ (v1.1-3), ‘gplots’ (v3.1.1), ‘pheatmap’ (v1.0.12) and ‘factoextra’ (v1.0.7). UHC was performed using the heatmap.2 function from the gplots package in R, which implements complete-linkage clustering based on Euclidean distances between rows (transposed scaled data) and columns (samples). Default parameters were used: Euclidean distance metric, complete linkage agglomeration, and no explicit reordering beyond dendrogram constraints. Kaplan-Meier curves depicting survival data were generated in GraphPad Prism v9 using data obtained from the Gene Expression Omnibus (GEO) via the Kaplan-Meier Plotter ^6^. Specific GEO datasets used are GSE12945, GSE13294, GSE14333, GSE143985, GSE17538, GSE18088, GSE26682, GSE30540, GSE 31595, GSE33114, GSE34489, GSE37892, GSE38832, GSE39582, GSE41258 and GSE92921. All samples in each dataset were used, no restrictions were applied. Processed metabolomic and transcriptomic data were scaled individually using z-score normalisation (mean=0, standard deviation=1) before being integrated to create a single data matrix. Principal Component Analysis (PCA) was conducted in R Studio using packages ‘tidyverse’ (v1.3.1), ‘ggplot2’ (v3.3.5), ‘factoextra’ (v1.0.7), ‘rgl’ (v0.108.3) and ‘plot3D’ (v1.4). Radar charts were created by scaling the data and plotting the average value for each variable in RStudio using packages ‘fmsb’ (v0.7.5) and ‘scales’ (v1.3.0). Leave-one-out cross validation (LOOCV) and corresponding ROC plots were created in R Studio using packages ‘tidyverse’ (v1.3.1), ‘dplyr’ (v1.0.9), ‘plyr’ (v1.8.7), ‘klaR’ (v1.7-1), ‘pROC’ (v1.18.5) and ‘caret’ (v6.0-93). The area under the ROC curve (AUC) was calculated using LOOCV predictions from a logistic regression model fitted via the ‘caret’ package in R, with class probabilities for the positive class processed by the pROC package's trapezoidal rule to integrate true positive rate against false positive rate across all thresholds. A probability of p<0.05 was considered statistically significant.

**Supplemental material references**

1. Ryan R, Gibbons D, Hyland JM, et al. Pathological response following long-course neoadjuvant chemoradiotherapy for locally advanced rectal cancer. *Histopathology* 2005;47:141–146.

2. Zukunft S, Prehn C, Röhring C, et al. High-throughput extraction and quantification method for targeted metabolomics in murine tissues. *Metabolomics* 2018;14:18.

3. Buckley AM, Dunne MR, Morrissey ME, et al. Real-time metabolic profiling of oesophageal tumours reveals an altered metabolic phenotype to different oxygen tensions and to treatment with Pyrazinib. *Sci Rep* 2020;10:12105.

4. Bolger AM, Lohse M, Usadel B. Trimmomatic: a flexible trimmer for Illumina sequence data. *Bioinformatics* 2014;30:2114–2120.

5. Fabregat A, Sidiropoulos K, Viteri G, et al. Reactome pathway analysis: a high-performance in-memory approach. *BMC Bioinformatics* 2017;18:1–9.

6. Győrffy B. Integrated analysis of public datasets for the discovery and validation of survival-associated genes in solid tumors. *The Innovation* 2024;5.

**Tables**

Table 1: Patient characteristics of cohort in which metabolomics of tissue was performed.

|  |  | **Cancer (*n*=32)** | **Non-Cancer (*n*=20)** |
| --- | --- | --- | --- |
| **Gender** | **Male (*n)*** | 20 | 12 |
|  | **Female (*n)*** | 12 | 8 |
| **Age** | **Mean (y)** | 63.8 | 60.95 |
|  | **Range (y)** | 48-89 | 40-81 |
| **Histology** | **Adenocarcinoma (*n)*** | 32 |  |
| **Differentiation** | **Poor-Moderate (*n)*** | 2 |  |
|  | **Moderate (*n)*** | 28 |  |
|  | **Well (*n)*** | 1 |  |
|  | **Unknown (*n)*** | 1 |  |
| **Clinical T stage** | **1 (*n)*** | 1 |  |
|  | **1/2 (*n)*** | 1 |  |
|  | **2 (*n)*** | 8 |  |
|  | **3 (*n)*** | 18 |  |
|  | **3/4 (*n)*** | 1 |  |
|  | **4 (*n)*** | 3 |  |
| **Clinical N stage** | **0 (*n)*** | 17 |  |
|  | **1 (*n)*** | 9 |  |
|  | **1/2 (*n)*** | 1 |  |
|  | **2 (*n)*** | 5 |  |
| **Pathological T stage^†^** | **0 (*n)*** | 3 |  |
|  | **1 (*n)*** | 4 |  |
|  | **2 (*n)*** | 7 |  |
|  | **3 (*n)*** | 10 |  |
|  | **4 (*n)*** | 1 |  |
| **Pathological N stage^††^** | **0 (*n)*** | 20 |  |
|  | **1 (*n)*** | 4 |  |
| **Treatment received** | **Neo-CRT (*n)*** | 16 |  |
|  | **Neo-RT (*n)*** | 2 |  |
|  | **Neo-CT (*n)*** | 1 |  |
|  | **Surgery only (*n)*** | 7 |  |
|  | **CT + RT only (*n)*** | 4 |  |
|  | **CT only (*n)*** | 1 |  |
|  | **Awaited (*n)*** | 1 |  |
| **TRS (Modified Ryan Score) (neo-CRT or neo-RT) ^†††^** | **0 (*n)*** | 3 |  |
|  | **1 (*n)*** | 7 |  |
|  | **2 (*n)*** | 5 |  |
|  | **3 (*n)*** | 1 |  |

**^†^**Pathological T stage available for *n*=25 patients only. **^††^**Pathological N stage available for *n*=24 patients only. **^†††^**TRS available for *n*=16 patients only, receiving either neo-CRT or neo-RT. Abbreviations; y, years; T stage, tumour stage; N stage, nodal stage; neo, neoadjuvant; CRT, chemoradiation therapy; RT, radiation therapy; CT, chemotherapy; TRS, tumour regression score.

Table 2: Patient characteristics of cohort in which real-time metabolic analysis of tissue was performed.

|  |  | **Cancer (*n*=11)** | **Non-Cancer (*n*=12*)*** |
| --- | --- | --- | --- |
| **Gender** | **Male (*n*)** | 5 | 6 |
|  | **Female (*n*)** | 6 | 6 |
| **Age** | **Median (range) (y)** | 69 (47-78) | 41.5 (26–81) |
| **Clinical T stage^†^** | **2 (*n*)** | 3 |  |
|  | **3 (*n*)** | 6 |  |
|  | **4 (*n*)** | 1 |  |
| **Clinical N stage^†^** | **0 (*n*)** | 6 |  |
|  | **2 (*n*)** | 3 |  |
|  | **3 (*n*)** | 1 |  |
| **Differentiation Stage** | **Moderate-poor (*n*)** | 2 |  |
|  | **Moderate (*n*)** | 5 |  |
|  | **Well (*n*)** | 1 |  |
|  | **Awaiting (*n*)** | 3 |  |
| **Treatment received** | **Neo-CRT (*n*)** | 5 |  |
|  | **Surgery only (*n*)** | 3 |  |
|  | **CT only (*n*)** | 1 |  |
|  | **Unknown (*n*)** | 2 |  |
| **TRS (of neo-CRT patients)** | **0 (*n*)** | 2 |  |
|  | **1 (*n*)** | 1 |  |
|  | **2 (*n*)** | 2 |  |

**^†^**Clinical tumour stage and clinical nodal stage only available for *n*=9 patients. Abbreviations; y, years; clinical T stage, clinical tumour stage, clinical N stage, clinical nodal stage; neo-CRT, neoadjuvant chemoradiation therapy; CT, chemotherapy; TRS, tumour regression score.

Table 3: Patient characteristics of cohort in which transcriptomics of tissue was performed.

|  |  | **Cancer (*n*=31)** | **Non-Cancer (*n*=28)** |
| --- | --- | --- | --- |
| **Gender** | **Male (*n)*** | 23 | 14 |
|  | **Female (*n)*** | 8 | 14 |
| **Age** | **Mean (y)** | 63 | 58 |
|  | **Range (y)** | 48-83 | 28-81 |
| **Histology** | **Adenocarcinoma (*n)*** | 31 |  |
| **Differentiation** | **Poor-Moderate (*n)*** | 2 |  |
|  | **Moderate (*n)*** | 27 |  |
|  | **Well (*n)*** | 1 |  |
|  | **Unknown (*n)*** | 1 |  |
| **Pathological T stage^†^** | **0 (*n)*** | 4 |  |
|  | **1 (*n)*** | 5 |  |
|  | **2 (*n)*** | 6 |  |
|  | **3 (*n)*** | 7 |  |
|  | **4 (*n)*** | 1 |  |
| **Treatment received** | **NeoCRT (*n)*** | 15 |  |
|  | **NeoRT (*n)*** | 1 |  |
|  | **NeoCT (*n)*** | 0 |  |
|  | **Surgery only (*n)*** | 8 |  |
|  | **CT + RT (no surgery) (*n)*** | 4 |  |
|  | **CT only (*n)*** | 1 |  |
|  | **Unknown (*n)*** | 2 |  |
| **TRS (Modified Ryan Score) (neo-CRT or neo-RT) ^††^** | **0 (*n)*** | 4 |  |
|  | **1 (*n)*** | 6 |  |
|  | **2 (*n)*** | 5 |  |
|  | **3 (*n)*** | 0 |  |

**^†^**Pathological T stage available for *n*=23 patients only. **^††^**TRS available for *n*=15 patients only, receiving either neo-CRT or neo-RT. Abbreviations; y, years; T stage, tumour stage; neo, neoadjuvant; CRT, chemoradiation therapy; RT, radiation therapy; CT, chemotherapy; TRS, tumour regression score.


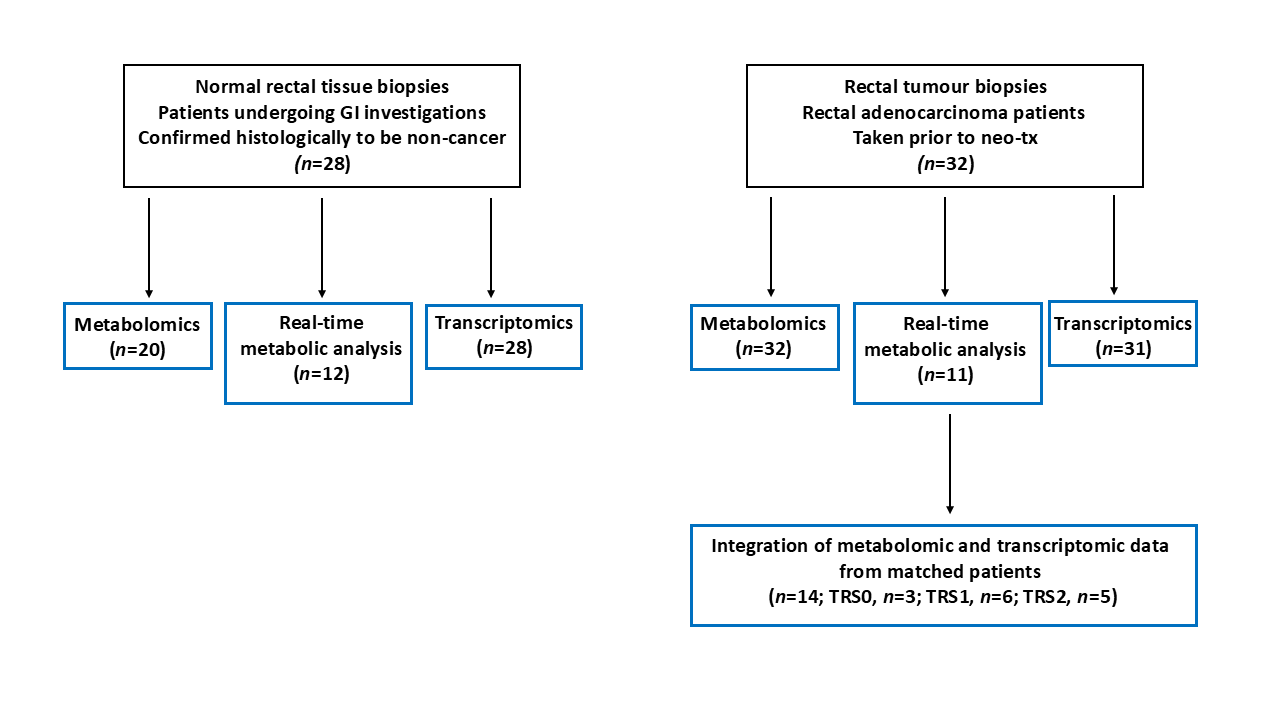
 **Supplementary Figure 1.** Flowchart of study samples and analysis.
